# Improvements in Glycemic Control and Depressive Symptoms Among Adults With Type 2 Diabetes: Retrospective Study

**DOI:** 10.2196/41880

**Published:** 2023-01-26

**Authors:** Aarathi Venkatesan, Gretchen Zimmermann, Kelly Rawlings, Claudia Ryan, Louise Voelker, Caitlyn Edwards

**Affiliations:** 1 Vida Health San Francisco, CA United States

**Keywords:** type 2 diabetes, digital health, diabetes intervention, diabetes, diabetic, health app, coaching, patient education, mobile health, mHealth, app-based, health coaching, hemoglobin A1c, HbA1c, depression, depressive, anxiety, mental health, glycemic control, diabetes management, health management, digital health intervention

## Abstract

**Background:**

The prevalence of diabetes remains high, with traditional lifestyle interventions demonstrating limited success in improving diabetes-related outcomes, particularly among individuals with diabetes-related mental health comorbidities. Digital health interventions provide the ability to ease the sustained and rigorous self-management needs associated with diabetes care and treatment. Current interventions though, are plagued by small sample sizes, underpowered pilot studies, and immense heterogeneity in program intervention, duration, and measured outcomes.

**Objective:**

Therefore, this work aimed to evaluate the effectiveness of a mobile health diabetes management program on measures of glycemic control in a high-risk population with type 2 diabetes (hemoglobin A_1c_ [HbA_1c_] ≥8.0%), utilizing a sample of 1128 participants who provided baseline and follow-up data. The sustainability of this change in glycemic control was examined in a subset of participants (n=455) at 6 months and 1 year following program enrollment. A secondary analysis examined changes in glycemic control among a subset of participants with self-reported mild-to-moderate depression at baseline.

**Methods:**

This study utilized a single-arm, retrospective design. Participants were enrolled in the Vida Health Diabetes Management Program. This app-based intervention utilized one-on-one remote sessions with a health coach, registered dietitian nutritionist, and/or a certified diabetes care and education specialist and structured lessons and tools related to diabetes management and self-care. Participants provided baseline (–365 to 21 days of program enrollment) as well as follow-up (at least 90 days following program enrollment) HbA_1c_ values. Two-tailed paired *t* tests were used to evaluate changes in HbA_1c_ between baseline and follow-up time points. The 8-item Patient Health Questionnaire and the 7-item Generalized Anxiety Disorder Scale were utilized to assess self-reported depressive and anxiety symptoms, respectively. Two-tailed paired *t* tests and linear regression modeling accounting for pertinent covariates were used to evaluate changes in mental health symptom acuity and their relationship with changes in glycemic control.

**Results:**

We observed a significant decrease in HbA_1c_ of –1.35 points between baseline (mean 9.84, SD 1.64) and follow-up (mean 8.48, SD 1.77; *t*_1127_=22.59, *P*<.001) among this large, high-risk sample. This decrease was sustained up to 1 year following program enrollment. Additionally, a significant relationship between improvements in depressive symptom acuity and improvements in HbA_1c_ was observed (β=–0.74, *P*=.03).

**Conclusions:**

This study demonstrates clinically meaningful improvements in glycemic control among participants enrolled in the Vida Health Diabetes Management Program. Additionally, this work presents one of the largest studied samples of participants enrolled in a digital health diabetes management program to date.

## Introduction

In the United States, 34.1 million individuals, or 13% of adults, are living with diabetes, and 88 million, or 34.5% of all US adults, are living with prediabetes [1]. Diabetes seldom develops in isolation, and there is a high prevalence of both physical and mental comorbidities with diabetes and prediabetes [2]. Physically, individuals with diabetes are at a higher risk of developing hypertension, dyslipidemia, and kidney disease. Mentally, individuals with diabetes are twice as likely to experience depression and 20% more likely to experience anxiety than the general population [3,4]. The development of effective and accessible interventions that address the polychronic nature of diabetes and related comorbidities is therefore an important next step in providing comprehensive diabetes treatment.

Clinical guidelines recommend that individuals diagnosed with diabetes should aim to maintain a hemoglobin A__1c__ (HbA__1c__) of less than or equal to 7.0% [5]. Reductions in HbA_1c_ of 1.0% over 10 years have been associated with a reduction in both diabetes-related deaths and microvascular complications [6,7]. Chronically elevated HbA_1c_ ≥8% can leave an individual more susceptible to the development of diabetes-related macro- and microvascular complications [8]. Meeting clinical guidelines, and reducing HbA_1c_, is achieved through a variety of diabetes self-management behaviors including but not limited to medical nutrition therapy, medication initiation and adherence, and monitoring of blood glucose values [9]. Diabetes is a chronic condition that requires a lifelong balance of complex self-care tasks by both the individual and their support system [3].

In an effort to account for the chronic self-care needs of diabetes treatment, much support has been found in incorporating mobile health (mHealth) technology into diabetes treatment. A commonly cited definition of mHealth is provided by the World Health Organization as “medical and public health practice supported by mobile devices, such as mobile phones, patient monitoring devices, personal digital assistants, and other wireless devices” [10]. With ever-growing access to smartphones and internet access worldwide, mHealth enables the delivery of health care services in a personalized, convenient, and cost-effective manner [11,12]. The growing ubiquity of mHealth technology ideally lends itself to supporting the initiation, development, and maintenance of behavior change, and as such, a number of apps designed for the education, treatment, and self-management of diabetes and related comorbidities are now available. These apps allow for the tracking of physical activity, nutrition, blood glucose levels, and medication or insulin dosage while also providing health feedback and education [13,14]. While many of these apps have demonstrated clinical effectiveness, with a recent meta-analysis suggesting the potential for 1.0% improvements in HbA_1c_ following use, not all mHealth interventions regarding the chronic management of diabetes and related-comorbidities have been created equally [15,16]. Published work is plagued by small sample sizes, underpowered pilot studies, and immense heterogeneity in program intervention, duration, and measured outcomes [17,18]. Alongside this, there is a paucity of rigorously designed published interventions on the use of mHealth apps to address the unique needs of populations who may be living with diabetes-related comorbidities.

For an individual with diabetes, the presence of one or more physical or mental comorbidities only exacerbates the magnitude of required self-management practices. Currently, no consensus exists on the methodology for the treatment of these conditions concurrently. A bidirectional link between depression and diabetes has been proposed with theories rooted in both physiology and psychology [19-23]. Physiologically, depression may be the result of the same neuroendocrine factors that influence diabetes progression, with suggested mechanisms including hypothalamic-pituitary-adrenal axis dysregulation and the chronic maintenance of a proinflammatory state [19-21]. Psychologically, the Lazarus and Folkman transactional model of stress and coping proposes a connection between mental health and diabetes in 2 different ways [22,23]: the first is that feelings of depression may stem from the diagnosis of diabetes itself, and the second is that the burden of the daily self-management needs of diabetes may result in the development of depression. Despite these theories, evidence supporting directionality is still lacking in the pathophysiology of these co-occurring conditions.

While mechanism directionality may be lacking, there is a plethora of literature on the interaction of simultaneous mental health and diabetes diagnoses on diabetes treatment [3,24,25]. A consistent relationship between depression and nonadherence to diabetes treatment regimen has been found, particularly as it pertains to missed medical appointments and adherence to dietary recommendations, medication, exercise, and glucose monitoring [26]. Additionally, the co-occurrence of these diagnoses can result in decreased overall health and increased economic burden of diabetes [27]. Based on this, a number of interventions have emerged aiming to address the impact of simultaneous treatment of mental health and diabetes, with the literature overall supporting potential clinical improvements following simultaneous treatment. A meta-analysis of randomized controlled trials evaluating the impact of coordinated multidisciplinary models of care for depression among adults with comorbid depression and diabetes revealed small-to-moderate effect sizes on HbA_1c_ that are comparable with pharmacological, psychological, and behavioral therapies [28]. With the goal of utilizing recent advancements in mHealth technology to provide accessible diabetes-based treatment with a focus on mental health, the Vida Health Diabetes Management Program was developed.

In this study, we evaluated the impact of the Vida Health Diabetes Management Program (referred to as Program throughout) on glycemic control (HbA_1c_) in a large population of individuals at high risk for diabetes-related complications (HbA_1c_ ≥8.0%). Vida Health is an app-based digital platform for chronic disease prevention and management that combines mobile technology and human-centered digital coaching to foster shared decision-making, goal setting, and accountability between provider and patient in daily diabetes and mental health self-care. We additionally evaluated two exploratory subaims. The first was to evaluate the longitudinal effectiveness of changes in glycemic control at 6 months and 1 year following Program enrollment. The second was to evaluate the impact of the Program on outcomes of depression and glycemic control among a sample of individuals with self-reported mild-to-moderate depression. We hypothesized that changes in glycemic control would both be maintained 1 year following Program enrollment, and that in a subsample of participants with both diabetes and depression, we would observe improvements in both depressive symptoms as well as glycemic control.

## Methods

### Ethics Approval

This study was approved by an independent institutional review board (Western Institutional Review Board), which waived informed consent as the study was identified as having minimal risk, because the data were fully anonymized before use in the analysis.

### Recruitment and Enrollment

Adults from a single payer client of Vida Health were recruited for this study through brochures, calling campaigns, and email announcements. Potential participants were offered the app-based Program as part of their medical insurance benefit. All participants enrolled in the study had smartphone or web-based access and were fluent in spoken and written English or Spanish. The Program was made available in both English and Spanish through professional translation services and employs bilingual providers. Participants accessed the Program through the Apple App Store or the Google Play Store and entered an invitation code unique to their insurance carrier for complementary Program usage. Next, participants completed a number of intake forms where they provided contact information, basic demographic information, and existing health conditions. Participants were excluded from study inclusion if they indicated the presence of chronic kidney disease stage 5, congestive heart failure classes III or IV, pregnancy, or breastfeeding.

Laboratory data pertaining to glycemic control (HbA_1c_) was provided by the insurance carrier at a monthly cadence utilizing protected and standardized data-sharing arrangements.

### Intervention Program

The Program is a digital health intervention designed to enable and empower individuals to manage their health through frequent provider interaction and rigorously designed multimedia content. The Program was designed under the self-determination theory [29], but employs a combination of techniques for implementation, including motivational interviewing, goal-setting, and self-monitoring. After completing the app intake, participants are paired with a certified health coach, a registered dietitian nutritionist, and/or a certified diabetes care and education specialist. All providers receive extensive evidence-based training on diabetes self-management and operate under a motivational interviewing framework to promote self-efficacy and autonomy for behavior change [30,31].

The Program from both the provider and participant perspective was designed to operationalize a complete feedback loop. Participants are offered weekly sessions with a provider for the first 12 weeks and then monthly thereafter as needed, at the discretion of the provider and the participant. The first interaction between provider and participant includes a detailed assessment to identify personalized user health goals and potential barriers to outcome success. Follow-up provider support is provided through on-demand in-app messaging and weekly remote video sessions, where providers and participants work to follow up on goal progress and resolve ambivalence to change. In between provider sessions, participants are encouraged to message their provider as often as needed and receive daily in-app content to provide education and support treatment goals. They are also provided with the option to explore all available in-app content for questions that may come up outside of provider sessions and daily content. All app usage is tracked by providers, and biometric data are assessed at each video session to allow for up-to-date data monitoring, interpretation, and pertinent adjustment of treatment as needed.

**Figure 1:**
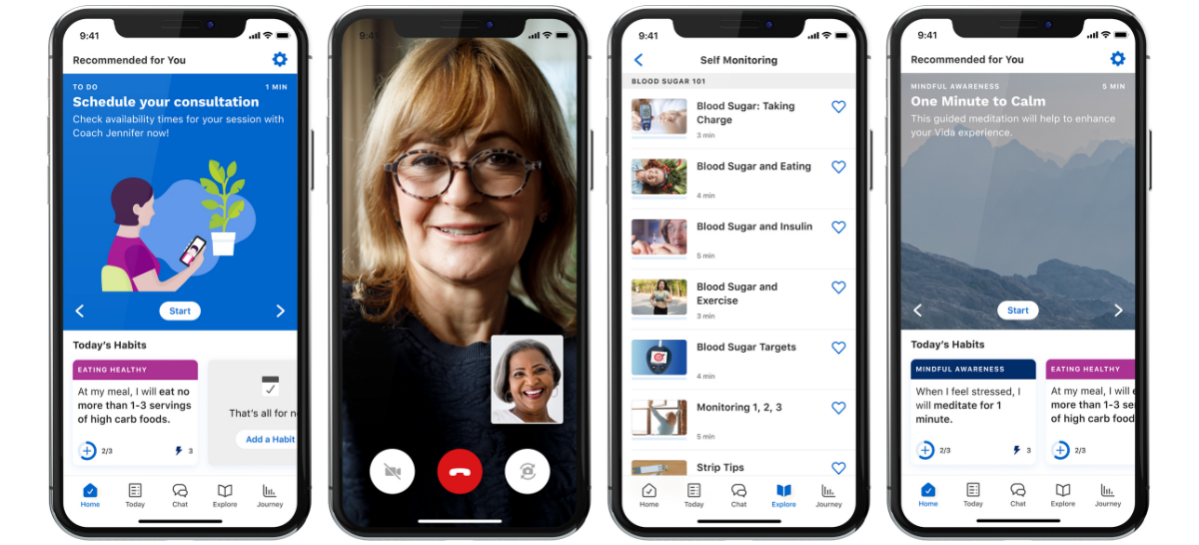
Screenshots of the Vida Health Diabetes Management Program.

Daily app content includes evidence-based structured lessons and multimedia content pertaining to self-monitoring behaviors as outlined in the Association of Diabetes Care and Education Specialists 7 Self-Care Behaviors framework as well as education on mindfulness, thought patterns, and the connections between mental and physical health [32,33]. Additionally, app-based content is based on cognitive behavioral therapy (CBT) principles, and participants complete lessons and activities designed to increase awareness of underlying thought patterns in order to promote the development of alternative, more adaptive thoughts. Alongside app-generated content, participants are encouraged to engage in a variety of logging activities: food logging, physical activity logging, and self-monitoring of blood glucose when appropriate. Additionally, the Program supports connections to a variety of commercially available cellular-connected blood glucose meters and continuous glucose monitors. Screenshots of the Vida Health user interface are provided in Figure 1.

### Mental Health Outcomes

Given the co-occurrence of mental health concerns with diabetes, all participants received mental health screenings for clinical depression and generalized anxiety. Depressive symptoms were assessed using the clinically validated 8-item Patient Health Questionnaire (PHQ-8) [34], and anxiety symptoms were assessed using the 7-item Generalized Anxiety Disorder Scale (GAD-7) [35]. The acuity of depressive symptoms for the PHQ-8 was defined using the following standard scoring cut-offs: 0-4 for asymptomatic/minimal, 5-9 for mild, 10-14 for moderate, 15-19 for moderately severe, and ≥20 for severe. The acuity of anxiety symptoms for the GAD-7 were defined using 0-4 for asymptomatic/minimal, 5-10 for mild, 11-16 for moderate, and ≥17 for severe. Participants who indicated severe acuity on either the PHQ-8 or the GAD-7 were referred to licensed care navigators for follow-up support. Completion of these assessments, while encouraged, was not mandatory. Both the PHQ-8 and the GAD-7 questionnaires were administered in the app automatically at program start, at week 6, at week 12, and every 3 months thereafter for up to 1 year. Additional assessments could also be sent at any time by the Program provider, at their discretion. Participants with baseline scores indicative of mild-to-moderate depression or anxiety were given the option of concurrently working with a licensed mental health professional in a 12-week structured CBT program.

### Statistical Analysis

The primary outcome measure for this study was HbA_1c_. HbA_1c_ was measured in clinical laboratories, and data were made available by the payer client. Baseline HbA_1c_ was defined as the laboratory test closest to Program start, measured between 1 year before (–365 days) to within 21 days after enrollment. Follow-up HbA_1c_ was defined as the HbA_1c_ assessment completed closest to 90 days within a minimum of 90 and a maximum of 365 days post Program start. For 13 participants, HbA_1c_ baseline laboratory data results were >14. In these instances, we confirmed the Logical Observation Identifiers Names and Codes test description as HbA_1c_ and the result unit of measurement as percent of total hemoglobin. Alongside the main analyses, a sensitivity analysis was conducted excluding these 13 individuals to account for their contribution to reported outcomes.

A number of individuals completed longitudinal assessments of HbA_1c_ following Program start. This subset of individuals was utilized to analyze the potential sustainability of changes in HbA_1c_ following enrollment into the Program. Three time periods were utilized for this analysis: the predefined baseline period (–365 to 21 days), a 3-6 month period (90 to 182 days), and a 7-12-month period (183 to 365 days).

A two-tailed paired *t* test was used to assess change in HbA_1c_ from baseline. A repeated measures ANOVA with the measurement period as a within-subject factor was used to analyze the sustainability of changes in HbA_1c_. A series of post hoc pairwise comparisons of means were conducted to evaluate HbA_1c_ changes between each measurement window.

A subanalysis was conducted among individuals who self-reported a score indicative of mild-to-moderate depression, had follow-up responses within 6 months on the PHQ-8, and had longitudinal laboratory provided HbA_1c_ values. Clinically meaningful changes in depression outcomes were defined as changes in self-reported symptom acuity. An example of a shift in acuity is moving from self-reported moderate depression symptoms at baseline to mild depression symptoms at follow-up. Two-tailed paired *t* tests were conducted between individuals who did and did not demonstrate improvements in symptom acuity to examine the relationship between reductions in mental health outcomes and glycemic control. Ordinary least squares regressions were also employed utilizing glycemic control as a dependent variable and the binary classification of acuity reduction (reduction=1 and no reduction=0), as well as covariates identified to be related to acuity reduction at baseline.

## Results

### Participant Characteristics

**Figure 2:**
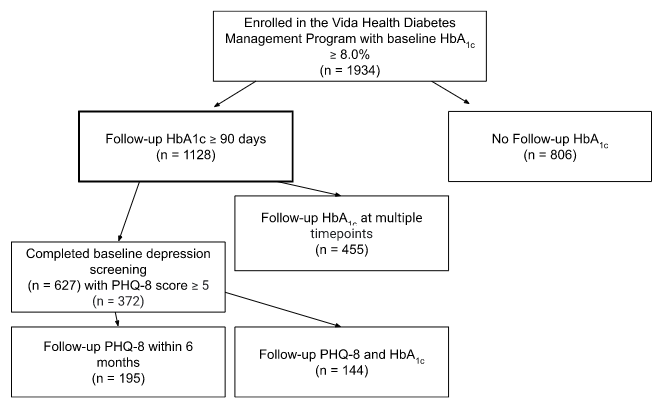
A flow chart demonstrating cohort identification. HbA_1c_: hemoglobin A_1c_; PHQ-8: 8-item Patient Health Questionnaire.

In all, 1934 participants with a baseline HbA_1c_ ≥8 were enrolled into the Program. Of those enrolled, 58.3% (1128/1934) provided follow-up laboratory HbA_1c_ values, and 40.3% (455/1128) of participants with follow-up data provided multiple laboratory HbA_1c_ values within a 1-year period. Additionally, 55.5% (627/1128) of participants with follow-up HbA_1c_ data completed a mental health screening, and 59.3% (372/627) indicated mild-to-moderate depression. Furthermore, 38.7% (144/372) of these individuals provided follow-up PHQ-8 data. Further information on the full cohort can be found in Table 1, and a schematic of participant flow can be found in Figure 2.

**Table 1:** Differences in age and baseline hemoglobin A_1c_ (HbA_1c_) as a function of gender^a^.

Characteristics		Participants (N=1128), n (%)	Age (years), mean (SD)	Baseline HbA_1c_, mean (SD)	Follow-up HbA_1c_, mean (SD)
**Gender**					
	Female	741 (64.5)	53.70 (9.84)	9.82 (1.66)	8.50 (1.78)
	Male	367 (31.86)	54.30 (8.98)	9.90 (1.58)	8.45 (1.78)
	Unidentified	20 (3.64)	54.40 (10.30)	9.52 (1.72)	8.82 (1.28)
Whole group		1128 (100)	54.10 (9.95)	9.84 (1.64)	8.48 (1.78)

^a^No significant differences were observed between gender for age, baseline, or HbA_1c_ value.

### Principal Results

**Figure 3:**
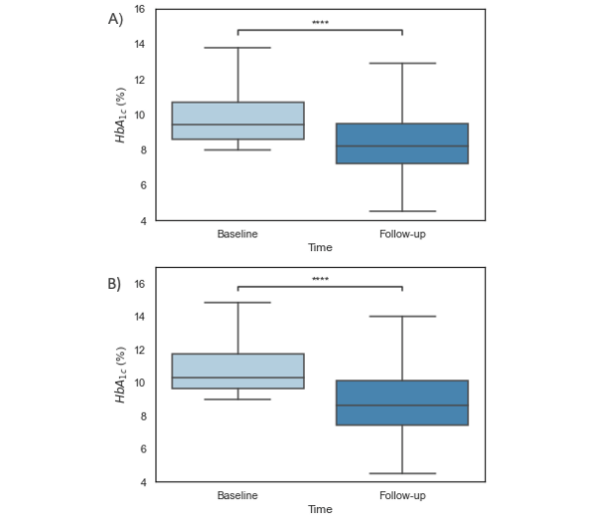
A box plot showing significant differences between baseline and follow-up HbA_1c_ among a sample of (A) 1128 participants with baseline HbA_1c_ ≥8.0% and (B) 687 participants with baseline HbA_1c_ ≥9.0% enrolled in the Vida Health Diabetes Management Program. HbA_1c_: hemoglobin A_1c_. **** Two-tailed paired *t* tests revealed significant differences between timepoints (*P*<.001).

Baseline HbA_1c_ measurements were completed on average –62.19 (SD 73.53) days prior to Program start, and follow-up HbA_1c_ measurements were completed on average 233.42 (SD 78.80) days from Program start. A two-tailed paired *t* test revealed a significant reduction in HbA_1c_ of –1.35 points between baseline (mean 9.84, SD 1.64) and follow-up (mean 8.48, SD 1.77; *t*_1127_=22.59, *P*<.001; Figure 3). Thirteen individuals reported an HbA_1c_ >14 at baseline. A sensitivity analysis was conducted excluding these 13 individuals, and we observed a significant reduction in HbA_1c_ of –1.31 points between baseline (mean 9.78, SD 1.56) and follow-up (mean 8.48, SD 1.77; *t*_1115_=22.29, *P*<.001). Among participants with baseline HbA_1c_ ≥9 (n=687), a reduction of –1.91 points was observed between baseline (mean 10.76, SD 1.48) and follow-up (mean 8.85, SD 1.93; *t*_686_=22.86, *P*<.001; Figure 3).

**Figure 4:**
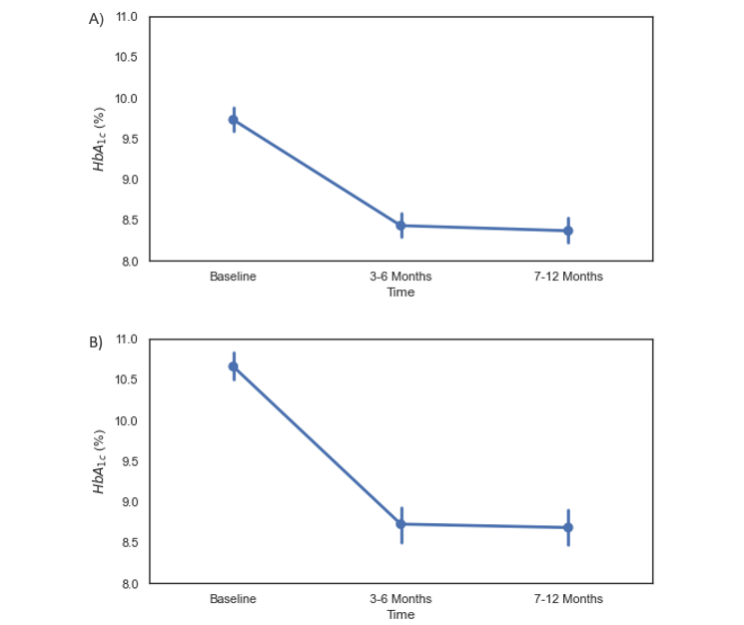
A figure showing significant changes from Baseline among participants with (A) Baseline HbA_1c_ ≥ 8.0% (n=455) at 3-6 months (-1.30 points, *P*<0.001) and 7-12 months (-1.36 points, *P*<0.001) and (B) Baseline HbA_1c_ ≥ 9.0% (n=265) at 3-6 months (-1.94 points, *P*<0.001) and 7-12 months (-1.98 points, *P*<0.001) following Program enrollment.

A number of individuals (n=455) had multiple assessments of HbA_1c_ following Program start. This subset of individuals was utilized to analyze the sustainability of changes in HbA_1c_ following enrollment into the Program. Three time periods were utilized for this analysis: the predefined baseline period (–365 to 21 days), a 3-6 month period (90 to 182 days), and a 7-12-month period (183 to 365 days). A repeated measures ANOVA with a Greenhouse-Geisser sphericity correction was conducted to assess the effect of measurement time point on HbA_1c_. We observed a significant effect of measurement time on HbA_1c_ (*F*_2,908_=162.30, *P*<.001). A post hoc pairwise comparison between measurement time points showed a significant decrease in HbA_1c_ of –1.30 points at 3-6 months following Program enrollment (mean 8.43, SD 1.64; Figure 4) from baseline (mean 9.73, SD 1.55; *t*_454_=14.0, *P*<.001). A reduction of –1.36 points was observed at 7-12 months (mean 8.37, SD 1.65; *t*_454_=14.6, *P*<.001). While this reduction was significantly different from baseline, the difference between follow-up time points was not statistically significant (*t*_454_=0.94, *P*=.35). In all, 265 participants with a baseline HbA_1c_ ≥9 had multiple assessments of HbA_1c_. Among these participants, we observed a significant effect of measurement time on HbA_1c_ (*F*_2,528_=174.05, *P*<.001). A post hoc pairwise comparison between measurement time points showed a significant decrease in HbA_1c_ of –1.94 points at 3-6 months following Program enrollment (mean 8.73, SD 1.79; Figure 4) from baseline (mean 10.66, SD 1.41; *t*_264_=14.7, *P*<.001). A reduction of –1.98 points was observed at 7-12 months (mean 8.68, SD 1.76; *t*_264_=15.0, *P*<.001). While this reduction was significantly different from baseline, the difference between follow-up time points was not statistically significant (*t*_264_=0.44, *P*=.66).

### Mental Health Subanalysis Results

An exploratory subanalysis was conducted among individuals who completed a baseline assessment and reported a score indicative of mild-to-moderate depression. At baseline, 372 (59.3%) out of 627 individuals reported a score indicative of mild-to-moderate depression. Of these 372 individuals, 195 (52.4%) completed at least one follow-up assessment within 6 months of Program enrollment (mean 116.71, SD 51.60 days). The average reduction in PHQ-8 score was –3.68 (SD 5.83) points (*t*_194_=8.63, *P*<.001) from baseline (mean 11.10, SD 3.82) to follow-up (mean 7.42, SD 5.43). In all, 119 (61%) out of 195 participants experienced a downward shift in depression acuity (movement from a higher symptom acuity at baseline to a lower symptom acuity at follow-up).

Of those with mild-to-moderate depression, 216 (58.1%) out of 372 participants also had scores indicative of mild-to-moderate anxiety. Among those who reported having mild-to-moderate anxiety, 118 (54.6%) out of 216 individuals completed at least one follow-up assessment within 6 months of Program enrollment (mean 117.09, SD 52.93 day). The average reduction in GAD-7 score was –2.94 (SD 4.90) points (*t*_117_=6.52, *P*<.001) from baseline (mean 9.71, SD 3.47) to follow-up (mean 6.77, SD 4.83). In all, 65 (55.1%) out of 118 participants experienced a downward shift in anxiety acuity.

To examine the relationship between clinically significant improvements in depressive symptom acuity and glycemic control, we employed an ordinary least squares regression. To test our hypothesis that clinically significant improvements in depressive symptom acuity would lead to subsequent improvements in glycemic control, we examined follow-up HbA_1c_ in the time period immediately following our 6-month mental health outcomes window, which was 7-12 months after Program enrollment. Paired *t* tests revealed that individuals who did experience a significant reduction in depressive symptom acuity reported higher depression baseline scores (difference: 1.20; *t*_142_=2.17, *P*=.03), but did not have higher baseline HbA_1c_ values (*P*=.23). Individuals who experienced a shift in depression acuity demonstrated a higher reduction in HbA_1c_ during this time period (mean –1.47, SD 1.97) compared to those who did not experience a significant shift in acuity (mean –0.77, SD 1.88; *t*_142_=–2.08, *P*=.04). Regression analyses with HbA_1c_ change as the dependent variable revealed that reduction in depressive symptom acuity was predictive of change in HbA_1c_ (β=–0.74, *P*=.03), while baseline depression score was not (β=0.08, *P*=.08).

## Discussion

### Principal Findings

This study evaluated the impact of the Vida Health Diabetes Management Program on glycemic control (HbA_1c_) in a population of over 1000 individuals at high risk for diabetes-related complications (baseline HbA_1c_ ≥8.0%). Following the Program, we observed a clinically and statistically significant decrease in HbA_1c_ of –1.35%, with this reduction sustained at 6 months and 1 year following Program enrollment. Additionally, among a subset of individuals who indicated experiencing mild-to-moderate depression at baseline, improvements in depressive symptom acuity were associated with an increased reduction in HbA_1c_.

Typical mHealth interventions are notoriously plagued by small sample sizes and underpowered pilot studies. With an included sample of 1128 participants, to the authors knowledge, this study is one of the largest published samples of an mHealth continuous care program aimed at improving measures of glycemic control to date. This large sample allowed for a robust assessment of the impact of our intervention on glycemic control. A recent review and meta-analysis suggests that reductions in HbA_1c_ of 1.0% are commonly found in mHealth programs designed to treat and assist with the management of diabetes, with the noted caveat that this work may not be entirely generalizable due to the small samples of the included studies [16,36]. In this study, our intervention not only exceeded this expectation and provided support for a similar reduction observed in our previous work but provides novel evidence that the observed reductions greater than 1.0% were sustained up to a year following Program enrollment.

For an individual with diabetes and at high risk of developing diabetes-related complications, a sustained reduction in HbA_1c_ of the observed –1.35% can have profound impacts on health outcomes. A 0.9 point reduction has been associated with a reduction in the risk of cardiovascular disease and all-cause mortality by between 10% to 20% in patients with type 2 diabetes [37]. While multiple systematic reviews highlight the potential to achieve these results as early as 3-6 months (the recommended time frame for follow-up HbA_1c_ testing [38]) following treatment initiation or program enrollment, fewer studies have provided follow-up data to support the maintenance of these results. This is concerning, as the purpose of Diabetes Self-management Education and Support (DSMES)–based digital programs such as the Vida Health Diabetes Management Program is to “give people with diabetes the knowledge, skills, and confidence to accept responsibility for their self-management” [9]. While quantifiable metrics of self-care behaviors were not included in the 1-year follow-up in the present work, the maintenance of a reduction in HbA_1c_ of –1.30% points at 12-month follow-up is indicative of engagement in self-care behaviors to support glycemic control at this time. Future work is needed to fully understand the DSMES activities most associated with sustained glycemic control among individuals with diabetes.

Diabetes is associated with a number of related physical and mental co-occurring health-related diseases, including depression. In this current sample of adults with type 2 diabetes, more than half (59%) of individuals who completed a baseline assessment of depression self-reported a baseline score indicative of mild-to-moderate depression. This data support that there is a clear need for treatment options that address the co-occurrence of physical and mental health conditions during diabetes treatment. While directionality cannot be assumed from these findings, it is possible that improvements in depressive symptoms may be driving a portion of the observed improvements in glycemic control in this sample. Symptoms of depression can impact everyday behavior including sleep, eating habits, appetite, fatigue, and physical activity, with improvements in depressive symptoms linked to improvements in these everyday behaviors. These symptoms are particularly concerning as they pertain to diabetes treatment, as effective and chronic engagement in the rigorous self-management needs of diabetes care is crucial to treatment success. This requires an abundance of personal motivation and willingness to engage in the behavior change process, yet often individuals experiencing depression note lack of motivation as a depressive symptom [39]. It is possible that the 61% of individuals who experienced a decrease in depressive symptoms were better able to engage in the self-management behaviors required for diabetes treatment, resulting in improvements in glycemic control.

Support for this theory can be gleaned from our previous work examining the impact of CBT on depressive symptoms in a medically complex adult population [40]. In this work, individuals who experienced improvements in depressive symptoms achieved significantly greater weight loss at a 9-month follow-up compared to individuals with moderate depression who did not demonstrate improvements in depressive symptoms (4.5% body weight loss compared to 1.5%). These results, alongside the findings presented here within, highlight the potential differences in physical health outcomes able to be achieved when addressing the obstacle of mental health. While future work is needed to directly examine the directionality of these observed relationships, we believe this work to be beneficial in providing support for the simultaneous treatment of mental and physical health among those who may be facing the burden of a polychronic diagnosis.

### Limitations

While this study drew strength from its large sample size, it is not without limitations. The first is the utilization of a nonrandomized, observational design. While these findings are similar to our previous work conducted in similar samples [41], we are not able to draw causal inferences from these findings. The second is the lack of follow-up laboratory glycemic control data from this population. A number of reasons may have driven this drop in sample size at follow-up: high insurance turnover within the payer organization, systemic barriers to seeking treatment and obtaining laboratory testing during the COVID-19 pandemic, or a time lag in our claims-based data reconciliation system. Nonetheless, at least one follow-up laboratory value was available for over half of the study sample, and over one-third of our sample provided at least two follow-up glycemic control values. Third, while we assessed depression and generalized anxiety within the current work, it is possible that other factors may be driving the improvements in these constructs, such as improvements in diabetes distress. Future work aiming to understand the relationships between the observed improvements in depression and anxiety and diabetes distress is warranted. Finally, due to data sharing limitations, data regarding disease duration as well as previous attempts or programs utilized for diabetes management were not available. Previous work should aim to incorporate these variables into analyses to understand the impact they may have on diabetes management.

### Conclusions

In conclusion, this large-scale, retrospective study demonstrated clinically significant improvements in glycemic control that were sustained at 6 months and 1 year following program enrollment among a sample of adults with type 2 diabetes at high risk for diabetes-related complications. A subanalysis revealed an association between improvements in depressive symptoms and improvements in glycemic control among participants who reported mild-to-moderate depression at baseline. This data provides further evidence on the benefits of simultaneous mental and physical health treatment among individuals with diabetes.

## Abbreviations

CBT: cognitive behavioral therapy
DSMES: Diabetes Self-management Education and Support
GAD-7: 7-item Generalized Anxiety Disorder Scale
HbA_1c_: hemoglobin A_1c_
mHealth: mobile health
PHQ-8: 8-item Patient Health Questionnaire

## Data Availability

The data sets analyzed during the current study are available from the corresponding author on reasonable request.
